# Diphtheria

**Published:** 1885-05

**Authors:** John V. Allen

**Affiliations:** Phila.


### DIPHTHERIA.

JOHN V. ALLEN, M. D., PHILA.

Diphtheria is an infectious disease, and, from personal experience, not contagious; attacking the human family at any age, but more often between the ages of one and twenty years. It manifests a train of symptoms which are generally found in all diseases affecting or altering the quality of the blood. First, the initial chill, or chilly sensations intermingled with heat, pain in the throat, beginning generally on one side, and rapidly spreading or extending to the opposite; headache, backache, and a general tired, aching, weak feeling. This condition continues (without the simillimum be given early) twenty-four to forty-eight hours, when symptoms of a more severe type are manifest; the submaxillary lymphatic glands begin to swell, and if the disease be not checked at this period, infiltration of the entire connective tissue of the sub-maxillary region becomes involved, and the patient rapidly advances into a dynamic condition.

In this short paper my special object is to give my mode of treating this dreadful disease, and it is one that has proven to be exceedingly successful. The membrane found in this disease is albuminous in character, being an exudation from the blood, therefore no local applications whatever should be used; the principal solid components of the blood are albumen and fibrine; if you remove this membrane by douches, gargles, etc., you leave the blood in excess of fibrine, and there is rendered great liability to heart clots. Not only do you subject your patients to the risk of sudden death from heart-clot, but you perceptibly weaken the patient, as it is from the albuminous portion of the blood that we get our strength and vitality. In removing this membrane you leave a surface upon which more exudation will undoubtedly form, and thereby exhaust your patient.

My course of treatment is to discard all local measures. I examine the throat internally and externally, notice the locality of the membrane, color and peculiar shape, etc., inquire what part of the throat pain was first experienced. I next wish to know the aggravations and ameliorations of the throat symptoms, the directions of pain, and all peculiar sensations and feelings; then the concomitant symptoms, headache, backache, pains in limbs, etc., etc. After satisfying myself of the simillimum, I generally give one or two doses of the highest potencies I have, usually the CM, as I find I have quicker and better action from these potencies than the lower attenuations; when you find, on a subsequent visit, that the patient feels a little better, matters not how trifling, it is not advisable to give any more medicine, or you are sure to make the patient worse. In this, more than in other diseases, medicines in the higher preparations act most speedily and nicely; this I have experienced several times.

Nor do I think it advisable to give any alcoholic preparations in diphtheria, as they seem to act more as depressants than as stimulants, and invariably increase the temperature and pulse; beef tea, milk, all kinds of broths, and Horlich's dry extract of malt, dissolved in milk, are very beneficial and nutritive. These are the articles of diet I generally use. Never force a patient to eat when there is no appetite or craving for food, but wait, and the appetite will return if the proper homœopathic remedy be given.

The temperature of the room should be from 68° to 73° Fahr., well ventilated and lighted with the sun's rays. If disinfectants be used, I prefer Platt's chloride to all others, as it is perfectly odorless.

As to remedies, I find most often indicated Lach., Lyc., *Lac. can.*, Phytol., Bell., Bry., Rhus, Kali bich., *Arum triph.*, Merc-cyanide, Merc-prot-iod, Merc-bin-jod, Apis, Lachn., and Nit-ac. Some of the characteristic indications I will mention of each remedy.

LACHESIS.—Pains begin left side of throat and spread to right, worse from warm drinks, after sleep, and from least touch of the throat.

LYCOPODIUM.—Pains begin right side and spread to left, worse from cold drinks, and very irritable after sleep.

LAC CANINUM.—Begins generally left side and spreads to right; yellowish, gray, curdy deposit, looking as if it had been varnished; exudation diffuses like the lid of a pepper-box, and dry. (Pains move from side to side.)

PHYTOLACCA.—Dark brown color of pseudo membrane, worse right side; worse swallowing saliva; feeling of red hot ball in throat; worse least touch to neck; throat feels dry and large, like a cavern; the membrane hangs down in strings from the posterior nares; pains shoot into both ears on swallowing. Right side.

KALI-BICH.—The exudation extends into bronchia; croupy cough; expectoration tough, viscid; can be drawn into long strings; on swallowing pain shoots into ear of affected side; sharp shooting pains in left tonsil; better by swallowing; all throat symptoms worse on putting out the tongue.

ARUM TRIPH.—Throat sore; excoriated; cannot swallow; excessive acrid salivation; sensation of something hot in throat. (Phytol.) The corners of mouth, buccal cavity, and even the throat become sore and raw, emitting blood—so sore, in fact, that the patient refuses all food and drink, in consequence of the suffering occasioned by mastication or swallowing. The breath is very fetid, and the cavity of the mouth is covered with diphtheritic deposits and ulcers.

MERC-CYAN.—This is the only preparation of Merc. that produces the excessive weakness which is so characteristic of diphtheria; the tongue is dark red and almost black; very offensive breath; saliva thin and fetid; profuse epistaxis; glands swollen and cellular tissue of neck infiltrated.

MERC-PROT-JOD.—Membrane worse on right side; tenacious mucus in throat; sick, offensive odor; worse from warm drinks (Lach.); thick, dirty yellow coating at the base of the tongue; glands swollen.

MERC-BIN-JOD.—Affects left side more than right; worse from empty swallowing; throat sensitive to touch; absence of coating on base of tongue.

APIS MEL.—Great debility characterizes the case from the outset; small amount of pain accompanying intense and extensive inflammation; the membrane has a dirty gray color; the diphtheritic patches usually appear on the arches of the palate over the uvula; the uvula œdematous and elongated; thirstlessness; scantiness of urine; perspiration frequently breaks out and dries up; heat is very unpleasant to the patient, etc.

LACHNANTHES.—When we have stiff neck, and head drawn to one side in the course of diphtheria.

NIT-ACID.—Spreading ulcers in the mouth and throat; putrid smelling breath; swelling of sub-maxillary and parotid glands (Merc-prot-jod.); corroding discharge from nose; dry, barking cough; strong-smelling urine, like that of a horse; sore throat extending up into nose, with profuse, thin, purulent discharge.

I would like to draw attention to Dr. Wm. J. Guernsey's card repertory of throat diseases. It is an excellent little work, and something that the homœopathic profession have been in need of for some time. It is in pamphlet form, and can be carried in the physician's pocket visiting list. Every physician should have one with him when prescribing for this disease or any other trouble of the throat. I find by using this repertory I have no trouble in selecting the homœopathic remedy.

